# Are the integrin binding motifs within SARS CoV-2 spike protein and MHC class II alleles playing the key role in COVID-19?

**DOI:** 10.3389/fimmu.2023.1177691

**Published:** 2023-07-10

**Authors:** Marijan Gerencer, Liam J. McGuffin

**Affiliations:** ^1^ Retired, Vienna, Austria; ^2^ School of Biological Sciences, University of Reading, Reading, United Kingdom

**Keywords:** SARS CoV-2 spike protein, integrin binding motifs, COVID-19, deregulated coagulation, immune evasion, autoimmunity, MultiFOLD model

## Abstract

The previous studies on the RGD motif (aa403-405) within the SARS CoV-2 spike (S) protein receptor binding domain (RBD) suggest that the RGD motif binding integrin(s) may play an important role in infection of the host cells. We also discussed the possible role of two other integrin binding motifs that are present in S protein: LDI (aa585-587) and ECD (661-663), the motifs used by some other viruses in the course of infection. The MultiFOLD models for protein structure analysis have shown that the ECD motif is clearly accessible in the S protein, whereas the RGD and LDI motifs are partially accessible. Furthermore, the amino acids that are present in Epstein-Barr virus protein (EBV) gp42 playing very important role in binding to the HLA-DRB1 molecule and in the subsequent immune response evasion, are also present in the S protein heptad repeat-2. Our MultiFOLD model analyses have shown that these amino acids are clearly accessible on the surface in each S protein chain as monomers and in the homotrimer complex and bind to HLA-DRB1 β chain. Therefore, they may have the identical role in SARS CoV-2 immune evasion as in EBV infection. The prediction analyses of the MHC class II binding peptides within the S protein have shown that the RGD motif is present in the core 9-mer peptide IRGDEVRQI within the two HLA-DRB1*03:01 and HLA-DRB3*01.01 strong binding 15-mer peptides suggesting that RGD motif may be the potential immune epitope. Accordingly, infected HLA-DRB1*03:01 or HLA-DRB3*01.01 positive individuals may develop high affinity anti-RGD motif antibodies that react with the RGD motif in the host proteins, like fibrinogen, thrombin or von Willebrand factor, affecting haemostasis or participating in autoimmune disorders.

## 1 Introduction

The SARS CoV-2 induced COVID-19 has led to the greatest pandemic outbreak since the appearance of the H1N1 influenza virus in 1918. Despite the extensive research on the ability of SARS CoV-2 to trigger multiple pathological phenomena in infected hosts, like deregulated coagulation, hyper inflammation and autoimmune diseases, the many factors and mechanisms that are involved in these events are still unknown. An important role in virus infections have various integrins that are present on the target cells.

Integrins are heterodimeric transmembrane glycoproteins composed of α and β subunits that bind extracellular matrix, cell-surface, and soluble ligands. They serve as cell adhesion receptors for numerous ligands playing an important role in signalling processes during infection and inflammation as well as in immunity, cell adhesion, cell migration, angiogenesis and carcinogenesis (1–3). The RGD (arginine/glycine/aspartic acid) amino acid sequence is the most frequent motif that plays a key role in integrin binding, but other tripeptide motifs have been identified such as KGD, LDV, ECD and MVD (4).

However, integrins may serve as entry receptors for a variety of different viruses including, coxsackie adenovirus, human cytomegalovirus, foot-and-mouth disease virus, Kaposi’s sarcoma-associated herpes virus, adenovirus, human papillomavirus-16, Hantaviruses (Sin Nombre virus), rotaviruses, echovirus-1, and others (5). Furthermore, viruses such as HIV, herpes simplex virus, measles viruses and SARS CoV-2, use integrins as co-receptors for entry into the host cells, which may enhance virus infectivity and broaden the cell types or hosts susceptible to infection (6, 7). There are several integrin categories based on their ligand specificity: collagen-binding integrins: α1β1, α2β1, α10β1, and α11β1; RGD motif- recognising integrins: α5β1, αvβ1, αvβ3, αvβ5, αvβ6, αvβ8, and αIIbβ3; laminin-binding integrins: α3β1, α6β1, α7β1, and α6β4; and leukocyte-binding integrins: αLβ2, αMβ2, αXβ2, and αDβ2 (8).

## 2 SARS CoV-2 spike protein and integrin binding motifs

### 2.1 The RGD integrin binding motif

The virus membrane proteins expressing the RGD motif are the most common integrin ligands contributing to the target cell entry by viral pathogens. Accordingly, the RGD motif plays a key role in virus infectivity and/or pathogenicity. For example, the RGD motif in Epstein-Barr virus (EBV) protein BMRF-2 is critical in infection of oral epithelial cells with EBV upon binding to β1 integrin subunit (9).

Furthermore, it is known that the RGD motif binding integrins play a significant role in coagulation, innate immunity, inflammation and autoimmunity (6). The RGD motif within plasma coagulation proteins such as fibrinogen, thrombin and von Willebrand factor (vWf) as well as within endothelial cell adhesion proteins fibronectin, laminin and vitronectin, binds to target integrins that are present on cells involved in coagulation, cell adhesion and/or immunity. The binding of fibrinogen to glycoprotein IIb/IIIa (GPIIb/IIIa), also known as platelet integrin αIIbβ3, plays a central role in platelet activation, haemostasis and arterial thrombosis. Integrin αIIbβ3 is expressed at a high level in platelets and their progenitors, where it plays the central role in platelet functions (10). The two RGD motifs within fibrinogen alpha chain, and the carboxyl-terminus of the gamma chain represent the potential αIIbβ3 binding sites on agonist-activated platelets such as adenosine diphosphate (ADP). ADP is an important primary platelet agonist that induces the activation-dependent conformational change in integrin αIIbβ3, which allows the fibrinogen binding and subsequent platelet aggregation initiating the coagulation process (11).

It is known that S protein from SARS-CoV-1 and SARS CoV-2 binds to the extracellular domain of angiotensin-converting enzyme 2 (ACE2) to infect the epithelial cells (12, 13). However, other cell entry mechanisms of SARS-CoV-2 have been strongly suggested (14). The recently evolved SARS CoV-2 S protein RGD motif (aa403-405) within the receptor binding domain (RBD) has been proposed to bind platelet integrin αIIbβ3 (7, 15). The RGD motif is not present in the S proteins from any other known human or bat coronavirus, but the KGD motif (lysine/glycine/aspartic acid), which is located at position aa390-392 in the SARS CoV-1 S protein RBD (16), is also able to bind αIIbβ3 integrin (4). The KGD motif is present also in the S proteins from so-called “bat SARS-like” coronaviruses, such as, RaGT13, WVI1, RsSHC014, Rs3367, and HKU3 (17).

Besides fibrinogen, other acute phase proteins such as prothrombin and vWF are integrin αIIbβ3 ligands, and they also use the RGD motif to bind activated platelets (18). Fibrinogen also binds to integrin α5β1 on endothelial cells via the carboxyl-terminal RGD sequence (aa572-574) of α-chain with high affinity (Kd = 65 nM), which have broad biological implications (19, 20). Interestingly, Grobbelaar et al. (21) have shown that the addition of S1 protein to platelet-poor plasma induces structural changes of fibrinogen β and γ chains, complement C3, and thrombin as determined by mass spectrometry, which may cause dysregulation of the coagulation process and resistance to fibrinolysis in COVID-19 patients if it occurs *in vivo*. The RGD motif is also present in endothelial cell adhesion proteins fibronectin, laminin and vitronectin, which are playing an important role in cell migration and adhesion (5).

**Figure 1:**
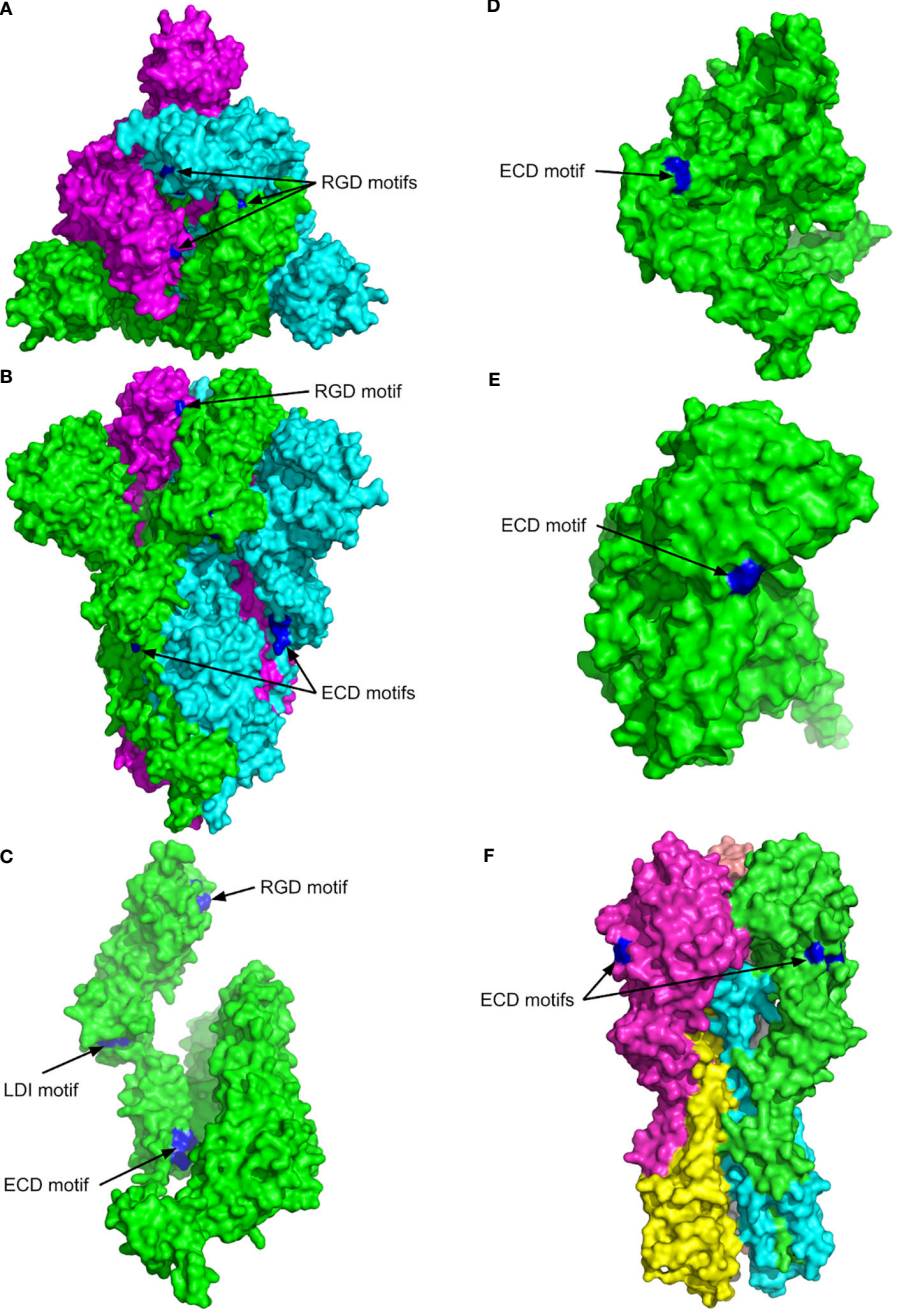
The 3D structures of SARS CoV-2 spike protein (P0DTC2, SPIKE_SARS2), ADAM17 metalloproteinase (P78536, ADAM17_HUMAN), zinc metalloproteinase-disintegrin-like acurhagin (Q9W6M5, VM3AH_DEIAC) and influenza haemagglutinin H1 (Q9WFX3). The surface views are shown with the key motifs labelled and highlighted in blue. **(A)** Top view of SARS CoV-2 spikes complex (PDB entry 6VXX). Chain A is shown in green, B in cyan, and C in magenta. **(B)** Side view of the SARS CoV-2 spike structure shown in **(A–C)** Chain A of the SARS CoV-2 spike structure. **(D)** MultiFOLD model of ADAM17 metalloproteinase (plDDT=0.833, pTM=0.760). The disordered residues from 1-33 and 711 onwards are removed for clarity. **(E)** MultiFOLD model of zinc metalloproteinase-disintegrin-like acurhagin (plDDT=0.912, pTM=0.856). **(F)** Side view of influenza haemagglutinin H1 complex (PDB entry 4GXX). Images are rendered using PyMOL (http://www.pymol.org/).

The previous studies have shown that endothelial injury is caused directly by the SARS CoV-2 (22), but the recent studies indicate that the expression of ACE2 was low or undetectable in non-COVID-19 pulmonary endothelial cells (23). The cells expressing low levels of ACE2 such as those in younger children (24), are more resistant to the SARS CoV-2 infection (25). Nader et al. (26) suggested that SARS-CoV-2 has a higher infection rate due to the ability of the S protein to bind αvβ3 integrin on vascular endothelium via the RGD motif. However, Beaudoin et al. (27) have performed the computational analysis of the protein structure and suggested that the binding of the RGD motif to integrins is very difficult in spite of the S protein unfolding and the subsequent conformational changes induced upon binding to ACE2 and that the S protein interacts with integrins independent of the RGD sequence. Our MultiFOLD model analysis of the experimental protein structure of the SARS CoV-2 S protein complex (UniProt ID - P0DTC2; PDB ID - 6VXX) shows that the RGD motif is partially accessible in each chain of the homotrimer complex (**Figure 1A**). But, if spike protein is able to bind integrins αIIbβ3 or αvβ3, the virus attachment to endothelial cells via S protein integrin binding motif(s) could lead to their infection and activation followed by dysregulation of the coagulation process and by excessive systemic inflammatory response. Thus, it is necessary to perform more extensive studies to clarify the possible role of the S protein RGD motif in COVID-19 pathogenesis.

Besides viruses, the malarial parasite *Plasmodium falciparum* uses the sporozoite surface Thrombospondin-related anonymous protein (TRAP; P16893) RGD motif (aa307-309) to interact directly with the host receptor integrin αvβ3, but it requires the contribution from vWF A domain (28–30). Furthermore, red blood cells (RBC) infected with *Plasmodium falciparum* exhibit erythrocyte membrane protein 1 (PfEMP1) that is synthesised during the parasite’s blood stage after the manifestation of clinical symptoms. PfEMP1 is also the ligand responsible for adhesion of RBC to endothelial cells via integrin αvβ3 that is thought to play a key role in the virulence of *Plasmodium falciparum*. These interactions can be inhibited *in vitro* by cyloRGDFV peptide, an antagonist of RGD-binding integrins (31).

An example of RGD motif-independent virus interactions with β3 subunit in αIIbβ3 and αvβ3 integrins is infection of endothelial cells with segmented negative-stranded RNA viruses, Hantaviruses NY-1 and Sin Nombre virus (32). After infection of endothelial cells, both viruses replicate primarily in the pulmonary endothelium causing Hantavirus cardiopulmonary syndrome (HCPS) with symptoms indistinguishable from those appearing in the initial phase of SARS CoV-2 infection in COVID-19 patients. Accordingly, the potential binding of SARS CoV-2 S protein to αIIbβ3 or αvβ3 integrin may induce identical pathological events in COVID-19 that are described in HCPS (33). Since the infection of endothelial cells with HCPS-associated Hantaviruses is inhibited by antibodies to β3 integrin subunit, the blocking of β3-integrins is the possible pharmacologic intervention in the initial phase of SARS CoV-2 infection.

The RGD motif-based peptide ligands have been tested in biomedical studies as low-molecular-weight integrin antagonists to treat the primary tumours and their metastases and for the control of inflammation, as well as for thrombosis inhibition (34–37). The RGD mimetic therapeutic antagonists, already on the market, have targeted integrins αIIbβ3 and αLβ2: Eptifibatide/Integrilin prevents platelet aggregation by inhibiting binding to fibrinogen in acute coronary syndrome and thrombotic cardiovascular events, and Lifitegrast prevents lymphocyte adhesion, thereby reducing T cell-mediated inflammation (38). Unfortunately, some current RGD motif-based anti-integrin drugs that are tested as agonists may trigger potentially fatal immune response and undesirable cell adhesion.

Therefore, it is necessary to perform further extensive studies that may confirm or reject the hypothesis that the S protein RGD motif plays an important role in COVID-19 pathology. If confirmed, it may bring solutions for more efficient COVID-19 therapy.

### 2.2 The LDV/I integrin binding motif

In addition to the RGD motif, other integrin-binding motifs like LDV (leucine, aspartic acid, valine) that binds to α4β1 integrin (very late antigen-4; VLA-4). This integrin is expressed on the cell surfaces of stem cells, progenitor cells, T and B cells, monocytes, natural killer cells, eosinophils, but not on neutrophils (39). The primary VLA-4 ligands are the vascular cell adhesion molecule-1 (VCAM-1) and the fibronectin connecting segment-1 (CS1) region during chronic inflammatory diseases, such as rheumatoid arthritis. VCAM-1 is an essential component of the endothelial cells activation cascade upon they express the VLA-4 integrin after stimulation by inflammatory cytokines, tumour necrosis factor alpha (TNF-α) and interferon-γ (IFN-γ). The VLA-4 integrin recognises the LDV motif within the sequence EILDVPST in the fibronectin CS1 region (aa2100-2107) promoting the inflammatory response and the movement of T lymphocytes to the site of inflammation (40–43).

The RBD within SARS CoV-1, CoV-2 and all bat SARS CoV-like spike proteins contains the EILDI sequence (aa583-587). Since Liu et al. (44) provided evidence that SARS CoV-2 S protein RBD also binds to broadly expressed integrin α5β1 with high affinity, the binding could be mediated by the S protein LDI motif, which enables SARS CoV-2 to infect ACE2 negative cells upon integrin activation after the initial ACE2 mediated infection of the lung epithelium. The LDI motif is also recognised by some non-RGD binding integrins, which indicates that besides the RGD motif an even broader range of integrins may be involved in the virus host-cell entry and in SARS-CoV-2 induced pathology (27, 45). Furthermore, the surface of the SARS CoV-2 S protein chain A complex shows the key accessible residues of the LDI motif (aa585-587) more clearly, and the LDI motif is also observed to be partially accessible in the homotrimer complex, **Figure 1C**.

Since the conservative amino acid replacement rarely results in dysfunction of the corresponding protein (46), it could be assumed that the conservative amino acid replacement of valine (V) with isoleucine (I) within SARS CoV-2 S protein RBD did not impact the LDI motif binding to α4β1 and/or α5β1 integrin. Similarly, it has been previously shown that the LDV/LDI switch within human immunodeficiency virus-1 gp120 could not change the binding affinity to target integrin, and even higher HIV-1 infectivity has been reported (47). The LDV/I motif encoded in the V2-loop of HIV-1 gp120 binds preferentially to integrin α4β7 (48), the lymphocyte receptor in the gut-associated lymphoid tissues, and the authors suggested that the LDI motif switch was linked to the successful epidemic dissemination of HIV-1 subtype C in South America, and to the other expanding non-B subtypes in Europe and Asia (49).

Therefore, it could be assumed that the S protein can act as an agonist and interferes with fibronectin binding to integrin α4β1 (VLA-4) or α5β1 promoting the inflammatory response by assisting in the movement of leukocytes to damaged tissue.

### 2.3 The ECD motif

Adamalysins, ADAM (A Disintegrin and Metalloproteinase) and ADAMTS (A Disintegrin and Metalloproteinase with Thrombospondin Motifs), are proteins characterised by their activity as zinc metalloproteinases and by disintegrin-like integrin receptor-binding domains (50, 51). Some of the ADAM proteins, like ADAM17, ADAM12 and ADAMTS13, have diverse roles in inflammation, vascular biology (fibrosis and thrombosis) and in virus entry into the cells. Particularly, ADAM17 (P78536) plays an important role in several human inflammatory autoimmune diseases, such as rheumatoid arthritis, multiple sclerosis, and systemic lupus erythematosus. Except its role in inflammation, ADAM17 is able to shed the surface platelet glycoprotein Ib alpha chain (GP-Ibα) allowing the platelet adhesion and plug formation at sites of vascular injury after binding to the vWF A1 domain on endothelium cells (52). The ADAM17 protein, also cleaves the TNF-alpha membrane-bound precursor to its active soluble form (53, 54). Furthermore, ADAM17, ADAM12 and ADAMTS13 have been found to be involved in COVID-19 pathogenesis (55). Since ADAM17 is found to be involved in the proteolytic processing of ACE2 (56), it may represent a novel molecular target for the drug development, as suggested by Schreiber et al. (57).

The integrin-binding amino acid sequence RGD within the disintegrin-like domain of many known ADAM proteins is employed in integrin-ligand interactions (58). However, some ADAMs, such as ADAM17, contain ECD (glutamic acid, cysteine, aspartic acid) or xCD motifs within the disintegrin-like domain that are involved in integrin-ligand interactions (59, 60). There is evidence that ADAM17 may act as one of α5β1 integrin ligands, and the integrin binding site is located within the disintegrin/cysteine-rich region that includes ECD motif (61, 62).

An example of the presumable ECD motif mediated ligand binding to target molecule was presented by Wang (63). Wang has shown that Acurhagin (Q9W6M5), an ECD disintegrin isolated from the snake *Agkistrodon acutus* venom, is able to induce apoptosis via caspase-3 activation in human umbilical vein endothelial cells *in vitro* after binding to αvβ3 integrin. Equally, purified αvβ3 integrin also binds to immobilised Acurhagin. These data suggest that the cell death induced by antagonist binding to αvβ3 integrin results in an apoptotic signal that is different from the apoptotic signal induced by programmed cell death (64). Most probably, the ECD motif plays an important role in binding to αvβ3 integrin that may activate caspase-3 and induce apoptosis as described previously for synthetic peptides containing the RGD motif (65).

Additionally, an ECD motif is present in S protein from SARS CoV-2 (aa661-663), SARS CoV-1 and bat SARS-like CoVs as well as in MERS S protein S1-C terminal domain (aa382-384), **Table 1**. The position of the ECD motif within the disulphide bond in each spike protein is very similar to the position of ECD motif in Acurhagin (**Figure 1A**), and the accessibility of the motif suggests that the S protein could also bind the αvβ3 integrin and cause caspase-3 activation and subsequent apoptosis in endothelial cells. The ECD motif is also present in pandemic influenza haemagglutinins H1 (UniProt ID: Q9WFX3) and H2 (UniProt ID: P03451), but not in H3 and H5 (**Table 1**). There is evidence that pandemic influenza A(H1N1) type causes more complications compared to other influenza virus subtypes, i.e., patients with A (H1N1) were admitted more often to the intensive care unit and died more often compared to A (H3N2) (66). However, the ECD accessibility, both in the S protein and in H1 as shown in **Figure 1**, means that the binding of both viruses to αvβ3 integrin on endothelial cells is possible and may induce apoptosis in infected endothelial cells. Therefore, if that assumption can be experimentally proven, it can explain the cause of the endothelial dysfunction associated with apoptosis after infection with SARS CoV or with influenza H1N1virus (67).

**Table 1:** The amino acid sequences within the disulfide bond adjacent to the ECD motif.

Proteins	Amino acid sequence	Position
Acurhagin	E**C**DPAEHCTGQSSECPADVFHKNGEPCLDNYGY**C**	466-499
Haemagglutinin HA1	**C**NIAGWLLGNPE**C**D	72-85
Haemagglutinin HA2	**C**SIAGWLLGNPE**C**D	70-83
Haemagglutinin HA3	**C**TLIDALLGDPH**C**D	80-93
Haemagglutinin HA5	**C**SVAGWLLGNPM**C**D	71-84
SARS Cov-2 S protein	E**C**DIPIGAGI**C**	661-671
SARS Cov-1	E**C**DIPIGAGI**C**	647-657
Bat SARS-like CoV WIV1	E**C**DIPIGAGI**C**	648-658
Bat SARS-like CoV Rp3/2004	E**C**DIPIGAGI**C**	633-643
Bat SARS-like CoV RaTG13	E**C**DIPIGAGI**C**	661-671
Bat SARS-like CoV 279/2005	E**C**DIPIGAGI**C**	633-643
Bat SARS-like CoV HKU3	E**C**DIPIGAGI**C**	634-644
Zaria bat CoV	E**C**QFSLGLDT**C**	688-698
Bat Cov HKU9	T**C**AMPIGNSL**C**	650-660
Rousettus bat coronavirus	V**C**EMPIGNSL**C**	666-676
Eidolon bat CoV/Kenya/KY24	D**C**SLLLGDSY**C**	647-657
MERS CoV (K9N5Q8)	E**C**DFSPLLSGTPPQVYNFKRLVFTN**C**	382-407
Bat CoV HKU4/2004	E**C**DFSPMLTGVAPQVYNFKRLVFSN**C**	387-412
Bat CoV HKU5/2005	E**C**DFTPMLTGTPPPIYNFKRLVFTN**C**	391-415
Bat CoV/133/2005	E**C**DFSPMLTGVAPQVYNFKRLVFSN**C**	388-412
Hypsugo bat CoV HKU25	E**C**DFSPMLTGTPPQVYNFRRLVFTD**C**	389-404
Bat Hp-CoV/Zhejiang2013	E**C**PFQSLINVTEATIPSPAFWRRHYVRN**C**	356-384

The amino acid sequences within disulfide bond adjacent to the ECD motif within in Acurhagin, influenza virus haemagglutinins and in spike proteins from SARS CoV-2, SARS CoV-1, bat SARS-like CoVs, MERS CoV and bat CoVs. The cysteine amino acids (C) within ECD motif forming the disulphide bridge (bold letters) in Acurhagin (Q9W6M5), influenza virus hemagglutinin HA1 (Q9WFX3), HA2 (Q67143), HA3 (O11283) and HA5 (P09345) and in the spike proteins from different human and mouse coronaviruses are shown. All amino acid sequences from the UniProt data base (17).

### 2.4 The accessibility of the RGD, LDIand ECD motifs

We have analysed the accessibility of RGD, LDI and ECD motifs within SARS CoV-2 S protein as well as the accessibility of such motifs in proteins that use these motifs to bind the target molecules using MultiFOLD analyses (68).

**Figure 1** shows the surface views of the experimental structures in the PDB with the best resolution and highest coverage for the SARS CoV-2 spike protein complex (UniProt ID - P0DTC2; PDB ID - 6VXX) and influenza haemagglutinin H1 complex (UniProt ID - Q9WFX3; PDB ID - 4GXX) (69) , and the MultiFOLD models of ADAM17 metalloproteinase (UniProt ID - P78536) and Acurhagin disintegrin (UniProt ID - Q9W6M5), which are confidently predicted (plDDT ⪞0.7 and pTM ⪞0.5, indicating that the predicted folds are likely to be correct). MulitFOLD is our new tertiary and quaternary structure modelling pipeline that has been independently verified to outperform both AlphaFold2 (70) and AlphaFold2-Multimer (68). The RGD motif in the SARS CoV-2 spike protein (aa403-405) is shown to be partially accessible in each chain of the homotrimer complex, **Figure 1A**. The side view of the SARS CoV-2 spike protein complex also shows that the ECD motif residues (aa661-663) are clearly accessible in each chain, **Figure 1B**. Furthermore, the surface of chain A of the SARS CoV-2 spike complex shows the key accessible residues of the LDI motif (aa585-587) more clearly, **Figure 1C**. The LDI motif is also observed to be partially accessible in the homotrimer. The ECD motif residues are shown to be accessible on the surface of both the ADAM17 metalloproteinase and the zinc metalloproteinase-disintegrin-like acurhagin MultiFOLD models (**Figures 1D, E**), as well as on the surface of the experimental structure of influenza haemagglutinin H1 complex (71) (**Figure 1F**). The top 5 predicted conformations for the targets in **Figures 1D, E** are shown in **Supplementary Figures 1A, B** respectively.

**Figure 2:**
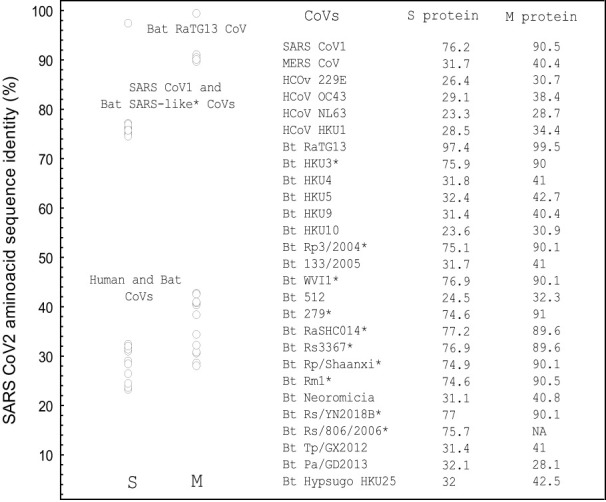
The amino acid sequence identity of the single spike and M proteins from different human and bat coronaviruses with the SARS CoV-2 spike (P0DTC2) and M ((P0DTC5) protein as determined by pairwise sequence alignment (%). NA, sequence not available. The alignments were performed using EMBOSS Needle algorithm (71, 72). *Bat SARS-like CoVs. S protein sequences: UniProt data base (17). Performed using STATISTICA, Statsoft.

Interestingly, besides the RGD motif, which is present only in the SARS CoV-2 S protein, the LDI and ECD motifs exist in SARS CoV-1 and all bat SARS-like CoVs that generally share about 75% amino acid sequence identity with the SARS CoV-2 S protein (**Figure 2**). An exception is the ECD motif, which is also present in the MERS CoV S protein and in some bat CoVs (**Table 1**). The pairwise sequence alignment of the SARS CoV-2 S and M protein with the S and M proteins from SARS CoV-1, bat SARS-like CoVs and human and bat corona viruses (CoVs), revealed about 75% identity with the S protein from SARS CoV-1 and bat SARS-like CoVs, except for bat RaGT13 CoV with 97.4%, whereas the identity level with known human and bat CoVs as well as with MERS CoV varied from 23.3% with human CoV-NL63 to 32.4% with bat CoV-HKU4. The amino acid sequence alignment regarding the M protein showed the similar results, but identity was generally much higher in comparison to the S protein, **Figure 2**.

## 3 The SARS CoV-2 S protein and autoimmunity

### 3.1 The SARS CoV-2 S protein and virus immune evasion strategy

Viruses often target the host’s innate immune system to bypass the immune response after infection using numerous evasion strategies (73, 74). An example of an efficient immune evasion is Epstein-Barr virus (EBV) gp42 protein that allows the persistent infection and survival of the virus in human HLA class II positive B cells. Besides its role as coreceptor in B cell infection, the EBV lytic-phase soluble protein gp42 is able to inhibit the T-cell recognition of antigenic peptides presented by HLA class I and class II molecules through steric hindrance that mediates the CD8 T-cell immunity evasion (75–77).

**Figure 3:**
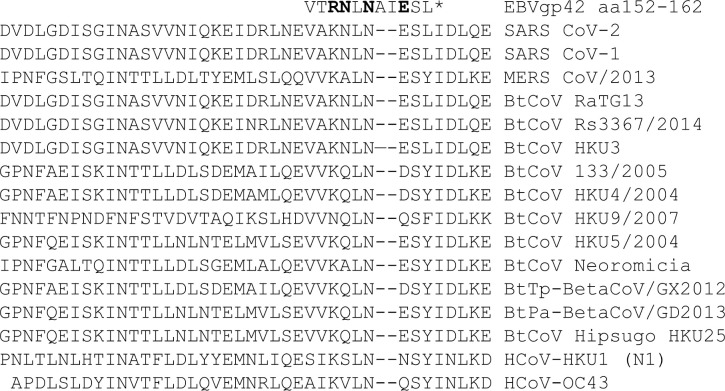
The alignments of EBV gp42 HLA-DRB1 binding domain sequence with the SARS CoV-2 and SARS CoV-1 S protein HR2 sequence as well with the HR2 sequences from different human and bat coronaviruses. The contact domain to the HLA-DRB1 b chain in the EBV gp42 protein including the binding amino acids: R154, N155, R157 and E160 (76) (* bold letters) aligned with the S protein HR2 amino acid sequence from SARS CoV-2, SARS CoV-1 and different human and bat coronaviruses.

**Figure 4:**
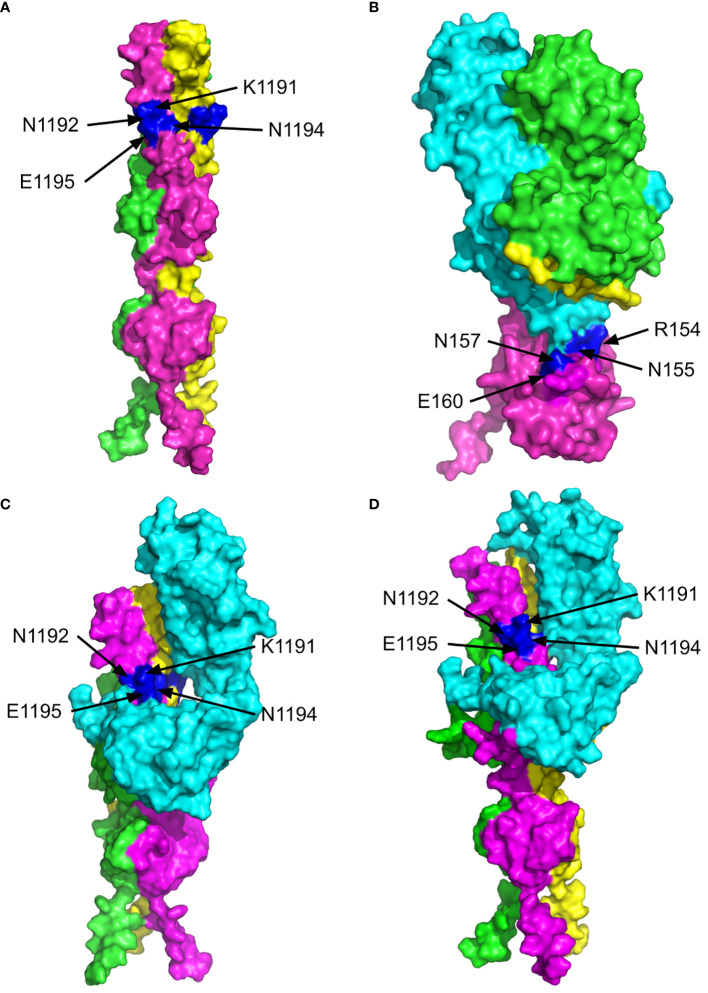
The 3D structures of the C-terminus of the SARS CoV-2 spike protein (P0DTC2, SPIKE_SARS2) and Epstein-Barr Virus (EBV) gp42 protein bound to the MHC class II Receptors HLA-DRB1 (P01911, DRB1_HUMAN) and HLA-DRB3 (P79483, DRB3_HUMAN) β-chains. The surface views are shown with the key residues labelled and highlighted in blue. **(A)** MultiFOLD model of the SARS CoV-2 spike (P0DTC2 - SPIKE_SARS2) homotrimer complex C-terminus region from residue 1194 onward (plDDT=0.751, pTM=0.599). **(B)** Crystal structure of EVB gp42 (magenta) bound to the HLA-DR1 complex (PDB ID - 1KG0). **(C)** MultiFOLD model of the SARS CoV-2 spike (P0DTC2 - SPIKE_SARS2) homotrimer complex C-terminus bound to HLA-DRB1 (P01911, DRB1_HUMAN) (plDDT=0.651, pTM= 0.497).**(D)** MultiFOLD model of the SARS CoV-2 spike (P0DTC2 - SPIKE_SARS2) homotrimer complex C-terminus bound to HLA-DRB3 (P79483, DRB3_HUMAN) (plDDT=0.627, pTM=0.485). Images of models were rendered using PyMOL (http://www.pymol.org/).

SARS CoV-2 has also developed multiple strategies to avoid appropriate immune response by reduction of fully protective immunity and by debilitation of long-lasting immune protection or by induction of an excessive immune response causing systemic inflammation and severe tissue damage after infection (78, 79). Similar to EBV gp42, heptad repeat-1 (HR1) and heptad repeat-2 (HR2) within the SARS CoV-2 S protein play an important role in virus-target cell interaction mediating viral fusion and host cell entry (80). Both, HR1 and HR2 have the most conserved sequences in the S protein, and HR2 is critical in viral entry (81). Interestingly, the amino acids within EBV gp42, R154, N155, R157 and E160 that are included in the binding to HLA class II molecules (76), are also present in SARS CoV-2 HR2, except the conservatively replaced amino acid R154 in EBV gp42 with K1191 (**Figure 3**), but this replacement does not influence their accessibility and the predicted 15-mer peptide binding score for HLA-DRB3*03.01 allele, **Figure 4**; **Table 2** (82). All of these amino acids are also conserved in HR-2 in SARS CoV-1 S protein and in HR2 from some bat SARS-like CoVs, **Table 2**. All contact amino acids are present in the predicted HLA-DRB3*03.01 strong binding 15-mer peptide RLNEVAKNLNESLID (core 9-mer peptide VAKNLNESL) in the previously detected B-cell epitope 1180EIDRLNEVAKNLNESLIDLQELGKYEQY1209 (83) within HR2, **Figure 3**.

**Table 2:** The prediction analysis of MHC class II binding peptides within SARS CoV-2 spike protein RBD and HR2.

MHC DRB alleles	15-mer peptide	Core 9-mer peptide	% Rank EL	
A) SARS CoV-2 RBD ^a^				
HLA-DRB1*03.01	ADSFVIRG**D**EVRQIA	IRGDEVRQI	1.83	7.80*
	DSFVIRG**D**EVRQIAP		0.98	8.75*
	SFVIRG**D**EVRQIAPG		0.73	8.81*
	FVIRG**D**EVRQIAPGQ		1.67	15.89*
	VIRG**D**EVRQIAPGQT		4.16	29.30*
HLA.DRB3*01.01	ADSFVIRG**D**EVRQIA	IRGDEVRQI	1.54	13.81*
	DSFVIRG**D**EVRQIAP		0.79	10.90*
	SFVIRG**D**EVRQIAPG		0.62	9.22*
	FVIRG**D**EVRQIAPGQ		1.52	18.94*
	VIRG**D**EVRQIAPGQT		4.11	35.51*
B) SARS CoV-2 HR2 ^a^				
HLA-DRB3*03.01	RLNEVAKNLNESLID	VAKNLNESL	1.79
	LNEVAKNLNESLIDL	VAKNLNESL	2.49
HLA-DRB3*02.01	RLNEVAKNLNESLID	VAKNLNESL	3.77
HLA-DRB3*01.01	RLNEVAKNLNESLID	VAKNLNESL	21.58

A) The predicted HLA-DRB1*03.01 and HLA-DRB*01.013 alleles binding peptides in the receptor binding domain of the SARS CoV-2 and Omicron variants BA.5 spike protein with mutation D405N*. B) The HLA-DRB3*03.01 strong binding peptides predicted in SARS CoV-2 HR2. The HLA-DRB binding peptides are predicted using the NetMHCIIpan-4.0 method for HighQ HLA molecules (82). The output results show the Eluted Ligand mass spectrometry (EL) % Rank scores for the SARS CoV-2 spike protein RBD (P0DTC2) and for Omicron variant BA.5 with mutation D405N (viralzone.expasy.org/9556). Threshold EL % Rank value for strong binders was 1, and for weak binders was 5. * EL % Rank values for Omicron BA.5 D405N mutation. The protein sequence data from UniProt data base (17).

**Figure 4A** shows the surface views of the MultiFOLD model of the C-terminus region from aa1158 of the SARS CoV-2 spike protein homotrimer (UniProt ID - P0DTC2, SPIKE_SARS2). The key residues (K1191, N1192, N1194, E1195) are shown to be accessible on the surface in each chain as monomers and in the homotrimer complex. The top 5 alternative conformations of the model in **Figure 4A** are shown in **Supplementary Figure 2A**. In **Figure 4B**, the EBV gp42 protein (magenta) is shown to be interacting with chain B (cyan) of the HLA-DR1 complex (UniProt IDs - P01903, P01911, P03437, P03205; PDB ID - 1KG0). The key residues of the gp42 (R154, N155, N157 and E160) are shown to be accessible on the surface at the site of interaction with HLA-DR1. The interaction site is shown in more detail in **Supplementary Figure 3A**. Furthermore, the top MultiFOLD models show that HLA-DRB1 and HLA-DRB3 bind with the SARS CoV-2 spike protein homotrimer at the sites of the key residues indicated in **Figures 4C, D**. The top 5 predicted conformations for the targets in **Figures 4C, D** are shown in **Supplementary Figures 2B, C** respectively, and the interactions are shown in more detail in **Supplementary Figures 3A, B** respectively.

However, it is not clear whether the binding of these amino acids to HLA-DRB1 molecule has the same role in immune response evasion and in virus fusion like soluble gp42 in EBV infection. It is necessary to confirm this assumption by further studies.

### 3.2 The SARS CoV-2 S protein and autoimmune manifestations

An immune response to some viruses may induce autoantibodies that cross-react with self-proteins and initiate an autoimmune disease. This mainly occurs by the mechanism of molecular mimicry, when viral and host proteins share structural similarities to an extent that results in an immune attack against autoantigens due to the breakdown of self-tolerance (84, 85). The autoimmune manifestations are associated with a wide spectrum of autoantibodies that are responsible for multisystem inflammatory syndrome, i.e., severe life-threatening disease. The homology regions amongst human and viral proteins may be the main cause of pathogen-induced autoimmunity, which induce severe health conditions in COVID-19 patients, or in patients with hypersensitivity pneumonitis (86). Unfortunately, some vaccines are able to stimulate formation of autoantibodies (87). Therefore, homology between human and viral proteins is a critically important issue in vaccine development that must be solved to prevent vaccine-induced autoimmunity. Since the great majority of vaccines used for immunisation against SARS CoV-2 are based on entire S protein or on its RBD domain, one may assume that the vaccine developers have not performed detailed studies on the potential occurrence of autoimmune phenomena after vaccination, especially after multiple boosters.

There is even more evidence that SARS CoV-2 infection deregulates the immune response against virus that often supports the development of autoimmune phenomena in COVID-19 patients (88). The deregulated immune response provoked by SARS-CoV-2 infection is characterised by hyper inflammation, coagulopathy and autoimmunity (89, 90). Although the majority of individuals infected with SARS CoV-2 are asymptomatic or with mild symptoms, a certain proportion of infected patients in intensive care units develop severe coagulopathy, hyperinflammation and autoimmunity (91). Unfortunately, multiple autoantibodies have been also reported after vaccination (92–94).

Autoantibodies are also detected in patients with post-COVID syndrome (95). When authors analysed sera from hundred patients with post-COVID syndrome and the presence of latent autoimmunity and poly-autoimmunity has been detected in 83% and 62% of patients, respectively. SARS-CoV-2 specific IgG antibodies were present in > 85% of patients, and they correlated positively with latent autoimmunity. Similar results presented Moody et al. (96).

However, it is not clear whether autoantibodies originate from a strong immune response to the S protein that potentiate a higher production of pre-existing autoantibodies or from an immune response against cross-reactive epitope(s) within the S protein or other SARS CoV-2 proteins. The most likely candidate that could induce cross-reactive antibodies is the RGD motif within the SARS CoV-2 S protein RBD. The RGD motif is known to support binding of coagulation proteins, such as fibrinogen and thrombin, to target integrin αIIbβ3 on platelets and their progenitors (97), but also binding of adhesion protein fibronectin to integrins exposed on endothelial cells (40).

### 3.3 The SARS CoV-2 S protein and autoimmunity in relation to HLA Class II alleles

We assume that the majority of autoimmune diseases that appear in COVID-19 and post-COVID syndrome may be linked to the single or multiple HLA alleles, and individuals with such an allele have and increased risk of developing an autoimmune disease (98). The HLA system plays key roles in the immune response, and predisposition to certain autoimmune disease is strongly associated with some of the HLA alleles that control the antigen presentation to T cells (99), and most frequently for ancestral haplotype-1 (AH8.1): HLA-A*01:01-C*07:01-B*08:01-DRB1*03:01-DQA1*05:01-DQB1*02:01. The HLA-DRB1*03 allele is a common allele in European populations and is the most frequent HLA allele included in AH8.1 (100, 101). AH8.1 has been found as a genetic factor responsible for susceptibility to several autoimmune diseases that are characterised by the low level of IgG2 type antibodies during the immune response to pathogens (102).

Candore et al. (103) have shown that individuals with A*01-B*08-DR3 haplotype have a defect in early T-cell activation and decreased production of IL2, IFNγ and IL12 as well as decreased natural killer cell activity (104). Moreover, the HLA-DRB3 allele, one of the most polymorphic HLA-DRB gene, is also linked to some autoimmune diseases (105) and to high responders against human platelet antigen-1a (HPA-1a) (106, 107).

The prediction of B- and T-cell epitopes within the known protein amino acid sequence by bioinformatics analysis and their confirmation by various bioassays *in vitro* is very important issue in vaccine development against the specific pathogen (108). After analysis, potential epitopes that could induce cross-reactive antibodies, i.e., epitopes that may cause an autoimmune response should not be included in the vaccine formulation. Vaccines that are not checked for such epitopes could cause autoimmune manifestations in vaccinated individuals, especially in individuals previously exposed to pathogen, e.g., SARS CoV-2 (91).

Recently published data on association of anti-SARS CoV-2 vaccine and myositis-related auto-antibodies that are reported after vaccination showed that HLA-DRB3.01 allele was one of the most prevalent alleles in affected patients (109). However, there are certainly other epitopes within SARS CoV-2 proteins, especially within S protein, which may be involved in triggering of an autoimmune response, both after infection and after vaccination, especially after multiple boosters.

Several autoimmune diseases are associated with HLA-DRB1*03.01 allele (110–112), but also with hyper immunoreactivity and rapid progression to the acquired immunodeficiency syndrome in HIV-1 infected patients (113). Furthermore, the HLA-DRB3* 01.01 allele indicates a predisposition to immunisation against human platelet antigen-1a (HPA-1a) in foetal and neonatal alloimmune thrombocytopenia (114) and its association with autoimmune disorders affecting the coagulation system during COVID-19 could be also assumed.

An initial step in the development of an adaptive immune response following infection with pathogens like SARS CoV-2 is the CD4+ T cell activation after recognition of antigenic peptides presented by HLA class II molecules on antigen presenting cells (APC). Activation of CD4+ T cells helps B cells to undergo isotype switching and develop antibodies with a higher affinity than those generated after T cell-independent activation (115, 116). Therefore, we assume that infected or vaccinated individuals could develop T-cell dependent antibody response to RGD motif within S protein RBD if it is included in an HLA class II binding peptide that is presented to B-cells by APCs.

We performed predictive analyses of HLA class II allele binding peptides within SARS CoV-2 S protein RBD for HLA-DRB1*03 and HLA-DRB3*01 alleles using improved NetMHCIIpan-4.0 method (117), which has been evaluated as an accurate method (118), such as NN-align and the IEDB consensus methods (119). The HLA class II allele binding peptide prediction analysis detected two identical strong binding and two weak binding HLA-DRB1*03:01 and HLA-DRB3*01.01 specific 15-mer peptides, which included the RGD motif in the core 9-mer peptide IRGDEVRQI, **Table 2**. It is known that the 9-mer core region within the 15-mer HLA class II binding peptide largely determines its binding affinity and specificity (119). The Eluted Ligand mass spectrometry (EL %) values of the SARS CoV-2 S protein RBD strong binding 15-mer peptides have shown high binding affinity to HLA-DRB1*03:01 and DRB3*01.01. Interestingly, the binding affinity of these peptides is significantly decreased and the predicted % Rank EL values were not in a positive range when the peptides included the D405N mutation in Omicron BA.2, BA.4 or BA.5 variants (viralzone.expasy.org/9556), **Table 2**.

### 3.4 Antibodies to the RGD motif in infection

The RGD motif is present in coagulation proteins such as fibrinogen and thrombin, as well as in other proteins involved in the coagulation process like fibronectin and vWF, and when developed, the RGD specific antibodies may interfere with the binding to target integrin αIIbβ3 on platelets and deregulate the coagulation process (97). According to the HLA class II binding peptide prediction results presented in **Table 2**, primarily the RGD motif specific antibodies may be expected in HLA-DRB1*01.03 or HLA-DRB3*01 positive individuals.

The antigenicity of the RGD motif has been evaluated by Yano et al. (120). They have shown that RGD motif remarkably enhanced peptide immunogenicity characterised on average by a 10x increase in antibody titre, when incorporated into the peptide sequence of a candidate vaccine. These data on the RGD motif antigenicity strongly support our prediction results for HLA class II binding peptides, **Table 2**.

Furthermore, Mohri et al. (121) have shown that anti-fibrinogen polyclonal antibody was capable of increasing the fibrinogen binding affinity to platelet αIIbβ3 integrin, which caused subsequent platelet activation and aggregation *in vitro*. Similarly, Althaus et al. (122) demonstrated that IgG fraction from severe COVID-19 patients was able to induce the FcγIIa receptor dependent procoagulant platelets that may contribute to the thromboembolic complications, but the antibody specificity and target epitope(s) were not determined in this study. However, it is not clear if these antibodies could affect the coagulation process and inflammatory response *in vivo*. One possibility is that fibrinogen/RGD antibody immune complexes bind to the platelet low-affinity receptor FcγRIIa via IgG Fc fragment (123, 124). This has been observed in infection with influenza virus H1N1 where the platelet activation occurs through stimulation of FcγRIIA receptor and thrombin generation (125). Therefore, we assume that the engagement of FcγRIIa receptor after antibody binding and triggering of the platelet activation and aggregation may be causally linked to the thromboembolic events in COVID-19 patients and in some vaccinees if they develop the RGD motif specific antibodies.

Interestingly, the RGD motif specific IgG antibodies have been previously detected in commercial intravenous immunoglobulin (IVIg) preparations (126). The authors used the RGD sequence-containing peptide AVTGRGDSPA to determine the antibody specificity. These antibodies are either innate antibodies or they originate from an immune response to the RGD motif present in the protein epitopes from other human viruses. Thrombotic events are also an increasingly recognised complication of treatment with IVIg preparation (127, 128), especially in patients with autoimmune disorders (129). Accordingly, since 2013 all commercial IVIg products have a safety warning about the potential risk of thromboembolic events after treatment.

Fibrinogen is also involved in inflammation as well in the promotion of autoimmune diseases such as rheumatoid arthritis (RA), chronic obstructive pulmonary disease, vasculitis and some other autoimmune disorders (97, 130, 131). Besides RA, ankylosing spondylitis and lupus nephritis are also associated with the disorder of the coagulation system and antibodies against citrullinated fibrinogen are frequently present in RA patients, and activation of the coagulation and fibrinolytic processes in the joints and in the circulation induce the inflammatory joint disease (132–135). It is possible that mainly SARS CoV-2 infected HLA-DRB1*03:01 and/or HLA-DRB3*01.01 positive patients develop RGD motif specific antibodies with higher affinity that react with fibrinogen or thrombin RGD motif and cause severe hypercoagulability complications during the coagulation process and fibrinolysis. Consequently, since fibrinogen and thrombin also play an important role in inflammation and autoimmunity (136), the deregulated coagulation process may also significantly contribute to inflammation and autoimmunity in COVID-19.

The role of HLA in COVID-19 pathogenesis is strongly supported by the findings showing a significant positive correlation of the HLA-A*: 01:01g-B*08:01g-C*07:01g-DRB1*03:01g haplotype with both COVID-19 incidence and mortality in the Italian population (suggestive of susceptibility), whereas haplotype HLA-A*02.01g-B*18.01g-C*07.01g-DRB1*11.04g showed a negative significant correlation (suggestive of protection) (137).

### 3.5 Antibodies to the RGD motif following vaccination

Long-lived high-affinity antibodies are the essential requirement for the successful vaccination strategies that could induce an effective defence against a particular pathogen. However, much attention has to be paid to possible autoimmune manifestations following vaccination, especially when infection with a target pathogen causes the appearance of autoantibodies (95).

The entire immune response to SARS CoV-2 vaccines depends on the presenting form of the S protein in a particular vaccine formulation. Therefore, antibodies to the S protein RGD motif immune epitope could be expected after vaccination with unmodified wild type S protein with the exposed RGD motif located on the loop surface of the RBD domain.

Rarely, following vaccination, some vaccinees experienced coagulation events that are very similar to what is described for heparin induced thrombocytopenia, including thrombosis in atypical sites caused by antibodies against platelet factor 4 (138–140). However, the existence of the RGD motif specific antibodies in COVID-19 patients and in vaccinees with thromboembolic complication needs to be proven, and the possible role of the HLA- DRB1*03 and DRB3*01 alleles in susceptibility to thromboembolic complications and autoimmune manifestations in SARS CoV-2 infected patients as well as in vaccinated individuals should be extensively investigated.

## 4 Discussion

We have discussed the hypothesis that the integrin binding motifs RGD, LDI and ECD within the SARS CoV-2 S protein bind to target integrins present on the host platelets and endothelial cells in the course of infection and play an important role in deregulated coagulation, inflammation, and autoimmune manifestations in COVID-19. On the basis of previous data and our analyses of protein structures using MultiFOLD models and multimers of known stoichiometry (68), as well as the prediction of HLA class II molecule binding peptides within the S protein RBD (82), we postulate that at least some of pathological manifestations in COVID-19 are linked to the S protein integrin binding motifs. The binding to target integrins may play an important role in coagulation (integrin αIIbβ3-RGD motif), cell adhesion (integrin α5β1-RGD/LDI motif), and inflammation (integrin α4β1/VLA-4-LDI motif) and induce deregulation of these processes or apoptosis (integrin αvβ3-ECD motif), **Table 3**. The fact that SARS CoV-2 Omicron variants BA.2, BA.4 and BA.5 with D405N mutation are less pathogenic with predominantly mild symptoms, support our assumption that the RGD motif plays very significant role in COVID-19. Bugatti et al. have shown that BA.5 variant with D405N mutation was not able to infect ACE2 receptor negative human lung microvascular cells *in vitro* due to the failed interaction between the S protein RGD motif and a*v*β3 integrin expressed on the cell membrane (141).

**Table 3:** The summary of the possible SARS CoV-2 spike protein motifs interaction with the cell membrane integrins in COVID-19 pathology.

Motif	Position	Target integrin (s)	Target cells/molecules	COVID-19 pathology
RGD	aa403-405	αIIbβ3 (7, 15) α5β1 (26) αvβ3 (27)	Platelets Endothelial cells Vitronectin	Coagulation disorders Infection of ACE2 negative cells
LDI	aa585-587	α4β1 (40–43, 46) α5β1 (44) α4β7 (48)	Fibronectin CS1 region Endothelial cells CD4+ lymphocytes	Uncontrolled inflammatory response
ECD	aa661-663	αvβ3 (64, 65, 141) α5β1 (61, 62)	Endothelial cells	Inflammation Apoptosis ADAM-17 activation

The most important references supporting the hypothesis are shown.

Furthermore, based on the prediction analysis of HLA class II binding peptides within the S protein RBD domain, an antibody response against the S protein RGD motif may be directed also against the RGD motif in the host plasma proteins that play an important role in coagulation process like fibrinogen or thrombin causing deregulation of their function. However, the most intriguing finding is the presence of amino acids within the S protein HR2 that are identical to those previously shown to be crucial in binding of the soluble EBV gp42 to HLA-DRB1 molecules, which plays an important role in EBV immune response evasion (76, 77). The 3D structures of the S protein N-terminus and the crystal structure of EVB gp42 bound to HLA-DR1 complex show that the key amino acids are clearly accessible. Accordingly, since these amino acids are also accessible in S protein HR2, we hypothesise that the SARS CoVs may use the same mechanism in the immune response evasion as EBV. However, all assumptions must be confirmed by further experimental research, and if confirmed, may lead to the development of new effective treatments and medications as well as to the safer and more effective S protein-based vaccines against SARS CoV-2 against upcoming coronaviruses. Also, an extensive research on the ability of the potential SARS CoV-2 vaccines to cause autoimmune manifestation in vaccinated individuals in the future, may avoid detrimental post-vaccination side effects like Graves’ disease or myopathies (92–94, 142, 143).

## Data availability statement

The original contributions presented in the study are included in the article/**Supplementary Material**. Further inquiries can be directed to the corresponding author.

## Author contributions

All authors listed have made a substantial, direct, and intellectual contribution to the work, and approved it for publication.
