# Supplementary material for: Are the integrin binding motifs within SARS CoV-2 spike protein and MHC class II alleles playing the key role in COVID-19?
Source: Front Immunol. 2023 Jul 10;14:1177691. doi: 10.3389/fimmu.2023.1177691 (PMC10364474; doi:10.3389/fimmu.2023.1177691)
Supplement: Supplementary file 1 [file DataSheet_1.docx]

*
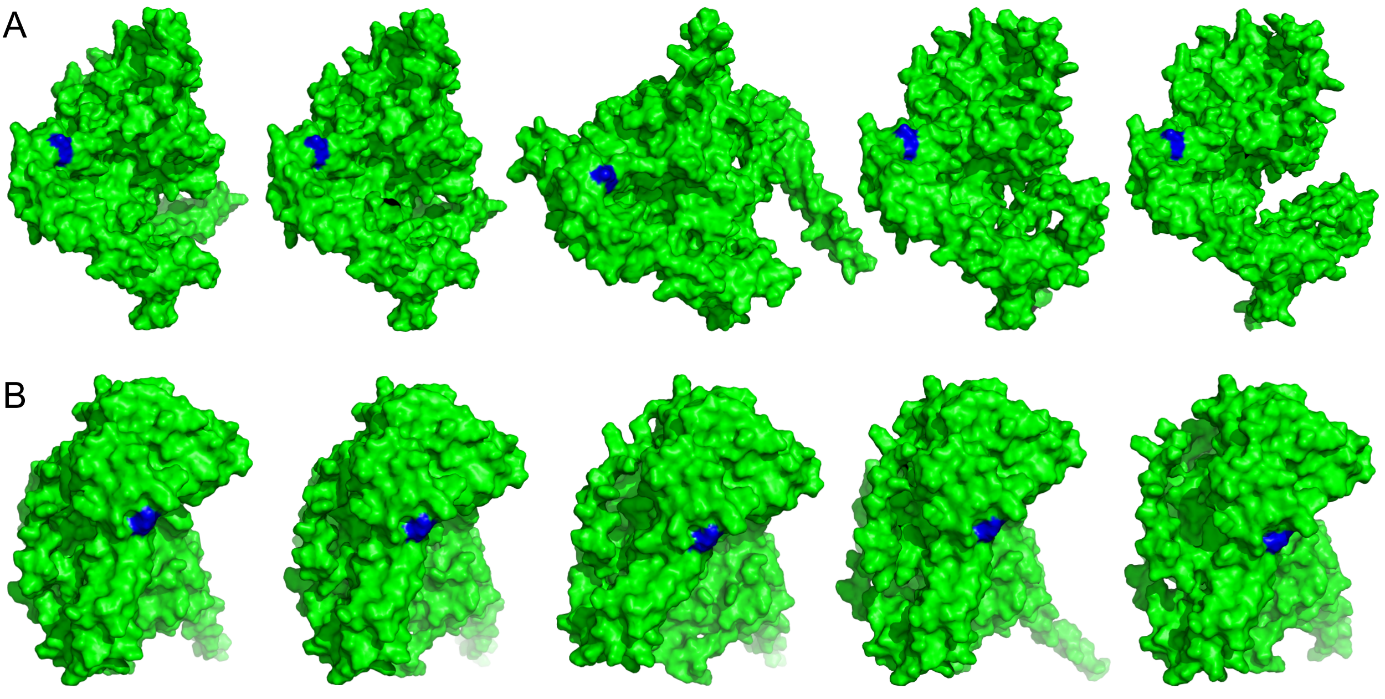
Supplementary Figure 1. Alternative MultiFOLD conformations for ADAM17 metalloproteinase (P78536, ADAM17_HUMAN) and zinc metalloproteinase-disintegrin-like acurhagin (Q9W6M5, VM3AH_DEIAC).*

A) The top 5 MultiFOLD models for ADAM17 metalloproteinase (P78536, ADAM17_HUMAN), from left to right: model1, plDDT=0.833, pTM=0.760; model 2, plDDT=0.800, pTM=0.707; model 3, plDDT=0.733, pTM=0.629; model 4, plDDT=0.742, pTM=0.619; model 5, plDDT=0.671, pTM=0.593. The models are available at: <https://www.reading.ac.uk/bioinf/MultiFOLD/MultiFOLD_view.php?time=9_39_37_495_19-4-2023_CASP_ALL_hdfa0clugggbi274&md5=hdfa0clugggbi274&targetname=P78536_ADAM17_HUMAN>.

B) The top 5 MultiFOLD models for zinc metalloproteinase-disintegrin-like acurhagin (Q9W6M5, VM3AH_DEIAC), from left to right: model1, plDDT=0.912, pTM=0.856; model 2, plDDT=0.906, pTM=0.839; model 3, plDDT=0.817, pTM=0.675; model 4, plDDT=0.828, pTM=0.651; model 5, plDDT=0.827, pTM=0.632. Models are available at: <https://www.reading.ac.uk/bioinf/MultiFOLD/MultiFOLD_view.php?time=9_44_45_761_19-4-2023_CASP_ALL_rl3k5uoqn4p02dol&md5=rl3k5uoqn4p02dol&targetname=Q9W6M5_VM3AH_DEIAC>.

Images are rendered using PyMOL (<http://www.pymol.org/>).

*
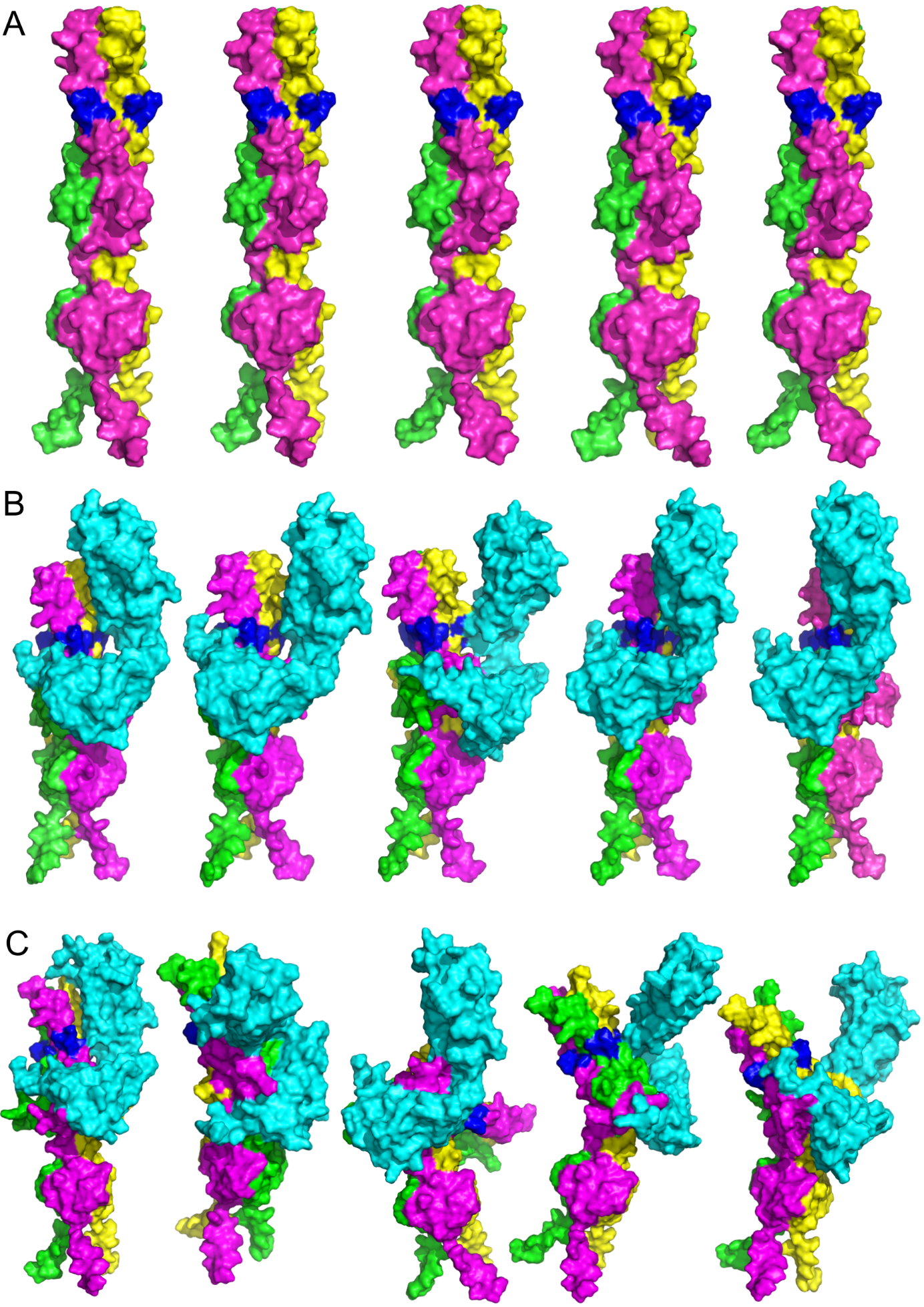
Supplementary Figure 2. Alternative MultiFOLD conformations for* *SARS CoV-2 spike (P0DTC2 - SPIKE_SARS2) homotrimer complex C-terminus, SARS CoV-2 spike (P0DTC2 - SPIKE_SARS2) homotrimer complex C-terminus bound to HLA-DRB1 (P01911, DRB1_HUMAN) and SARS CoV-2 spike (P0DTC2 - SPIKE_SARS2) homotrimer complex C-terminus bound to HLA-DRB3.*

A) The top 5 MultiFOLD models for SARS CoV-2 spike (P0DTC2 - SPIKE_SARS2) homotrimer complex C-terminus region from residue 1194 onward, from left to right: model1, plDDT=0.751, pTM=0.599; model 2, plDDT=0.721, pTM=0.565; model 3, plDDT=0.719, pTM=0.548; model 4, plDDT=0.746, pTM=0.546; model 5, plDDT=0.723, pTM=0.518. The models are available at: <https://www.reading.ac.uk/bioinf/MultiFOLD/MultiFOLD_view.php?time=18_3_16_11_18-4-2023_CASP_ALL_3nor3vh3ffvtn2hs&md5=3nor3vh3ffvtn2hs&targetname=SPIKE_SARS2_1194-end>.

B) The top 5 MultiFOLD models for SARS CoV-2 spike (P0DTC2 - SPIKE_SARS2) homotrimer complex C-terminus bound to HLA-DRB1 (P01911, DRB1_HUMAN), from left to right: model1, plDDT=0.651, pTM=0.497; model 2, plDDT=0.670, pTM=0.495; model 3, plDDT=0.695, pTM=0.485; model 4, plDDT=0.671, pTM=0.481; model 5, plDDT=0.659, pTM=0.475. The models are available at: <https://www.reading.ac.uk/bioinf/MultiFOLD/MultiFOLD_view.php?time=17_55_3_573_18-4-2023_CASP_ALL_l8knm01hb3hkq0sp&md5=l8knm01hb3hkq0sp&targetname=SPIKE_SARS2_1194-end_DRB1_HUMAN_trimmed>.

C) The top 5 MultiFOLD models for SARS CoV-2 spike (P0DTC2 - SPIKE_SARS2) homotrimer complex C-terminus bound to HLA-DRB3 (P79483, DRB3_HUMAN), from left to right: model2, plDDT=0.627, pTM=0.485; model 1, plDDT=0.648, pTM=0.500; model 3, plDDT=0.611, pTM=0.483; model 4, plDDT=0.646, pTM=0.479; model 5, plDDT=0.633, pTM=0.476. The models are available at: <https://www.reading.ac.uk/bioinf/MultiFOLD/MultiFOLD_view.php?time=17_58_46_736_18-4-2023_CASP_ALL_9hotv2vhbv32sn60&md5=9hotv2vhbv32sn60&targetname=SPIKE_SARS2_1194-end_DRB3_HUMAN_trimmed>

Images are rendered using PyMOL (<http://www.pymol.org/>).

**
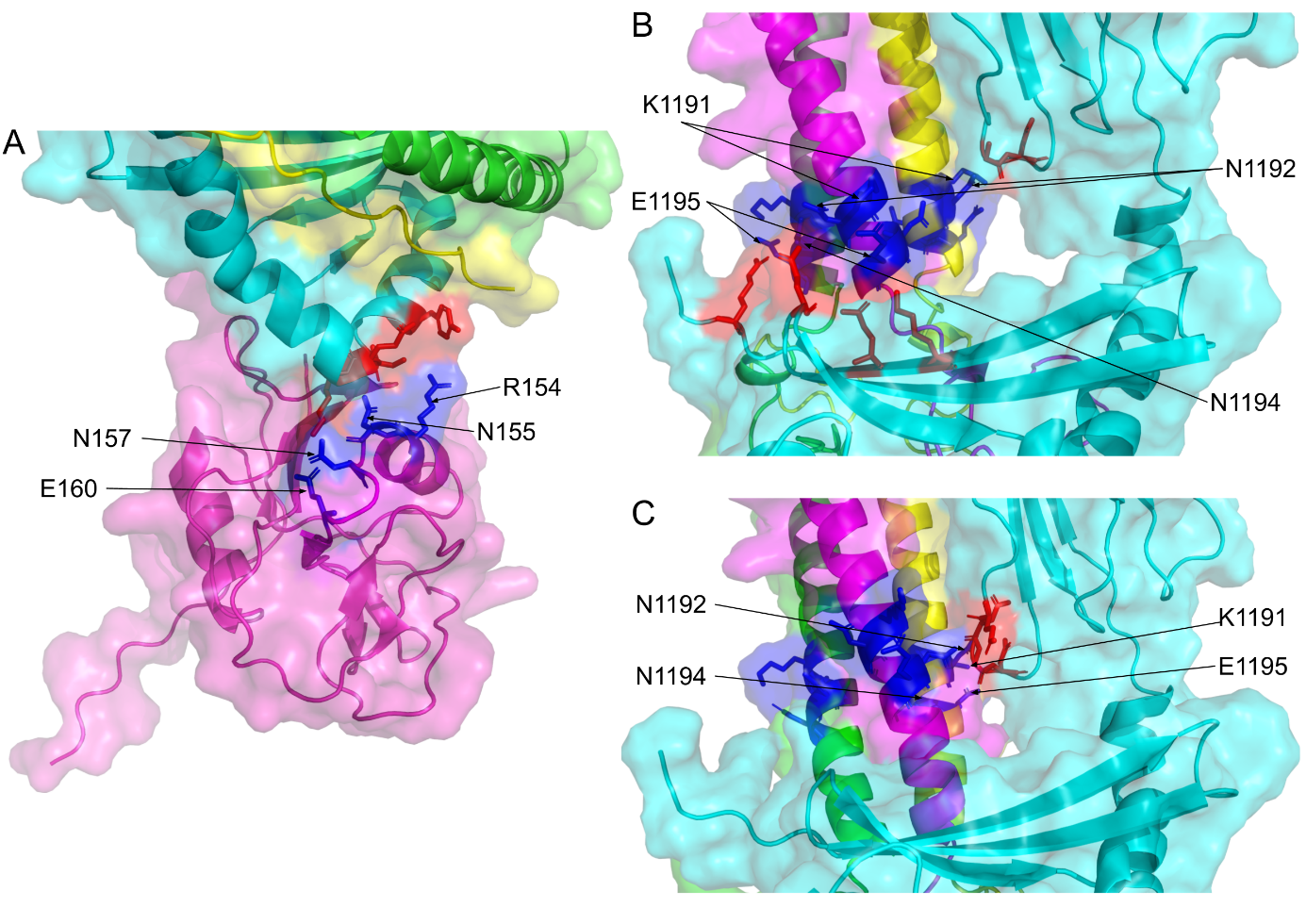
**

*Supplementary Figure 3. Close-ups of the interaction sites for the complexes shown in Figure 3.*

A) Interaction of EVB gp42 (magenta) with the HLA-DR1 complex (PDB ID - 1KG0) at the site of residues 89-95 (YWNSQKD) in chain B (cyan). Y89, S92, Q93, and K94 (red sticks) of 1KG0B are in contact with the key residues R154, N155, N157 and E160 (blue sticks).

B) Predicted interaction site for SARS CoV-2 spike (P0DTC2 - SPIKE_SARS2) homotrimer complex C-terminus bound to HLA-DRB1 (P01911, DRB1_HUMAN). The key residues from the spike protein (K1191, N1192, N1194, E1195; blue sticks) are in contact with residues R33, Q39, K41, Q63, G180, and D181 (red sticks) of HLA-DRB1.

C) Predicted interaction site for SARS CoV-2 spike (P0DTC2 - SPIKE_SARS2) homotrimer complex C-terminus bound to HLA-DRB3 (P79483, DRB3_HUMAN). The key residues from the spike protein (K1191, N1192, N1194, E1195; blue sticks) are in contact with residues Q178, N179, G180, and D181 (red sticks) of HLA-DRB3.

Images are rendered using PyMOL (<http://www.pymol.org/>).
